# Novel Aspects of the Physiology of Pregnancy in Domestic Ruminants

**DOI:** 10.3390/ani15182672

**Published:** 2025-09-12

**Authors:** Fuller W. Bazer, Thainá Minela, Gregory A. Johnson

**Affiliations:** 1 Department of Animal Science, Texas A&M University, College Station, TX 77843, USA; thaina.minela@ag.tamu.edu; 2 Department of Veterinary Integrative Biosciences, Texas A&M University, College Station, TX 77843, USA; gjohnson@cvm.tamu.edu

**Keywords:** ruminants, pregnancy, interferon tau, implantation, placentation, metabolism

## Abstract

Mammals exhibit unique and highly variable mechanisms for the establishment and maintenance of pregnancy. Ruminants (e.g., sheep, cows, and goats) have novel mechanisms whereby the conceptus (embryo and its extra-embryonic membranes) signals for the establishment of pregnancy and exhibits unique metabolic pathways favoring conceptus development. Embryos of ruminants reach the spherical blastocyst stage at 5 to 10 mm in diameter and then elongate rapidly to elongated filamentous conceptuses of greater than 250 mm as they make contact with the uterine luminal epithelium (LE) for implantation. During conceptus elongation the trophectoderm cells secrete interferon tau (IFNT), a novel pregnancy recognition signal for ruminants to ensure maintenance of a functional corpus luteum (CL) to secrete progesterone (P4) required for pregnancy. P4 induces uterine epithelia cells to express the endogenous Jaagsiekte Retrovirus (enJSRV) that may transactivate toll-like receptors 7 and 8 in the conceptus trophectoderm to induce secretion of IFNT, a classical viral–antiviral mechanism. IFNT silences expression of receptors for estradiol (E2) and oxytocin (OXTR), which abrogates the mechanism whereby oxytocin from CL and posterior pituitary would otherwise induce large pulses of prostaglandin F_2α_ (PGF) by uterine epithelia to cause regression of the CL and its secretion of P4. IFNT has another novel role in silencing expression of not only ESR1 and OXTR, but all classical interferon-stimulated genes in the uterine LE and superficial glandular epithelium (sGE), but with P4 increasing expression of genes for transport of nutrients such as glucose and arginine into the uterine lumen to support conceptus development. Ruminant conceptuses convert glucose to fructose, a novel hexose sugar that cannot be transported back to the maternal circulation. Fructose is converted to fructose-1-PO4 for metabolism, not via the pathway for glycolysis but via the novel fructolysis pathway uninhibited by low pH, citrate, or ATP as is the case for glycolysis. Thus, fructose and its metabolites support the pentose cycle, hexosamine biosynthesis pathway, one-carbon metabolism, and the citric acid cycle for all cells of the conceptus. Arginine is another key nutrient transported into the uterine lumen by the uterine LE/sGE in response to P4 and IFNT. Arginine is metabolized to generate nitric oxide, polyamines, and creatine, essential for conceptus growth and development, while enhancing production of IFNT as a novel pregnancy recognition signal, and upregulating expression of genes in the uterine LE/sGE for transport of nutrients. Fructose is the major hexose sugar supporting major metabolic pathways required for conceptus growth and development in ruminants.

## Simple Summary

Embryonic mortality in ruminant embryos occurs primarily during the pre- and peri-implantation periods of pregnancy when they must develop into spherical blastocysts and elongate rapidly to form a conceptus (embryo and its extra-embryonic membranes). The conceptus must then signal for the establishment of pregnancy, initiate contact with the uterine luminal epithelium for implantation, and begin converting glucose to fructose for further metabolism via fructolysis through the pentose cycle, hexosamine biosynthesis pathway, one-carbon metabolism, and tricarboxylic acid cycle. The conceptus trophectoderm secretes a novel pregnancy recognition signal, interferon tau (IFNT), perhaps in response to endogenous Jaagsiekte Retrovirus (enJSRV) expressed by uterine epithelia in response to progesterone (P4) that transactivates toll-like receptors 7 and 8 to induce secretion of IFNT. IFNT signaling to uterine epithelia silences expression of receptors for estradiol (E2) and oxytocin (OXTR) to abrogate the mechanism whereby oxytocin from corpora lutea and posterior pituitary induces luteolytic pulses of prostaglandin F_2α_ (PGF). IFNT, in concert with P4, also silences expression of classical interferon-stimulated genes in the uterine LE and superficial glandular epithelium (sGE) while increasing expression of genes for transport of key nutrients, such as glucose and arginine, into the uterine lumen. The conversion of glucose to fructose and then fructose-1-PO4 allows its metabolism via the fructolysis pathway not to be inhibited by low pH, citrate, or ATP. Arginine transported into the uterine lumen is required for production of nitric oxide, polyamines, and creatine, essential for conceptus growth and development. These key events in early pregnancy are essential for the survival of ruminant conceptuses.

## 1. Introduction

**Overview of the Estrous Cycle, Luteolysis, and Pregnancy in Ruminants**. Ruminants (e.g., cattle, sheep, and goats) are spontaneously ovulating, polyestrous mammals with recurring estrous cycles of 21, 17, and 20 days, respectively [1,2,3,4]. Domestic cattle (*Bos taurus*; *Bos indicus*) are not seasonal breeders, but sheep (*Ovis aries*) and goats (*Capra hircus*) are short-day breeders with regular estrous cycles from late summer through mid-winter. Endocrine regulation of the estrous cycle and pregnancy has been studied in greatest detail with sheep. In ruminants, Day 0 of the estrous cycle is the day of onset of sexual receptivity or estrus for mating and lasts about 30 h. Ovulation occurs about 30 h after estradiol (E2) from mature ovarian follicles leads to an estrogen-induced ovulatory surge of LH and FSH from the anterior pituitary. Metestrus, Days 1 to 4 of the estrous cycle, is characterized by luteinization of the theca and granulosa cells of the ovarian follicle under the influence of LH to form the corpus luteum (CL), which secretes progesterone (P4). Diestrus, Days 4 to 14 of the estrous cycle, is the period during which the CL reaches maximum size and secretes the greatest amounts of P4. Near the end of diestrus, receptors for P4 (PGR) in uterine epithelia are auto-downregulated by P4, allowing increases in expression of receptors for estradiol (ESR1) and E2-induced expression of receptors for oxytocin (OXTR) in uterine epithelial cells. This sets the stage for the pulsatile release of oxytocin (OXT) from the CL and posterior pituitary gland to act via OXTR to induce pulsatile secretion of luteolytic prostaglandin F2α (PGF) from uterine epithelial cells responsible for the structural and functional demise of the CL, known as luteolysis. The estrous cycle of ruminants is uterine-dependent as the uterus is the source of luteolytic PGF. During diestrus, P4 increases phospholipid stores and prostaglandin synthase in uterine epithelia required for the release of arachidonic acid from phospholipid stores by phospholipase A2 and its conversion by prostaglandin synthase 2 (PTGS2) and PGF synthase to PGF during late diestrus. Importantly, exposure of the uterus to P4 for 10 to 12 days downregulates PGR, which, in turn, allows ESR1 and OXTR expression by the uterine luminal (LE) and superficial glandular (sGE) initially and then the uterine glandular epithelium (GE) and stromal cells. If ewes are hysterectomized during the active life of the CL, luteolysis does not occur, and CL life span is prolonged to about five months, which is similar to the duration of a normal pregnancy.

**Figure 1:**
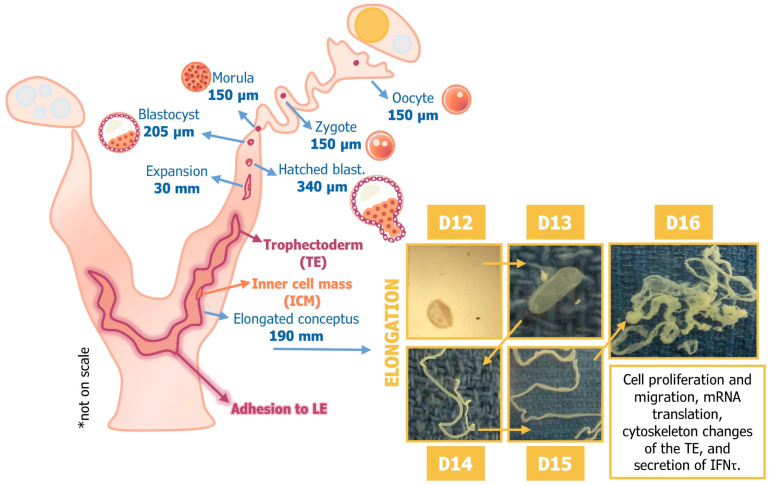
Conceptus development in sheep (see [3,4,5,6]). The ovine blastocyst hatches from the zona pellucida and undergoes morphological transitions from spherical to tubular and tubular to an elongated filamentous form. Elongation of the conceptus involves proliferation, migration, and cytoskeletal remodeling of trophectoderm cells, as well as secretion of interferon tau between Days 10 and 21 of gestation as the conceptus (embryo and extra-embryonic membranes) undergoes apposition and then adhesion to the uterine luminal epithelium (LE) during the peri-implantation period of gestation. * Please note that the drawing of the uterus, embryos and conceptuses is not to scale.

Secretion of ovine interferon tau (IFNT), measured as ng/mL uterine flush, begins on about Day 10 of pregnancy and increases as conceptuses undergo morphological changes from spherical (312 ng) to tubular (1380 ng) and filamentous (4455 ng) forms between Days 12 and 16 of gestation [4,5], as shown in Figure 1. IFNT silences transcription of *ESR1*. Therefore, ESR1-induced expression of the *OXTR* gene in uterine LE and sGE is abrogated to prevent development of the endometrial luteolytic mechanism requiring oxytocin-induced pulsatile release of luteolytic PGF. However, basal production of PGF is greater in pregnant than cyclic ewes due to continued expression of PTGS2 by the uterine LE/sGE. For most, if not all, actions of IFNT on the uterus, P4 is the permissive hormone required for the establishment and maintenance of pregnancy [6].

Uterine receptivity to implantation of the blastocyst and conceptus (embryo and its extra-embryonic membranes) is P4-dependent and preceded by loss of expression of PGR and ESR1 by uterine epithelia [7,8,9,10,11]. The loss of expression of PGR in uterine epithelial cells is a prerequisite for the implantation and expression of many interferon-stimulated genes by those cells in ewes. Thus, P4 likely acts via PGR-positive stromal cells to increase expression of what are termed progestamedin(s) in ewes, especially fibroblast growth factor 10 (FGF10) and hepatocyte growth factor (HGF), that exerts paracrine effects on uterine epithelia and the conceptus trophectoderm via their respective receptors, FGFR2IIIb and HGFR, encoded by the oncogene met (MET). Ovine uterine LE and sGE lack PGR and do not express classical interferon-stimulated genes (ISGs) such as signal transducers and activators of transcription (STAT1 and STAT2) and interferon regulatory factor 9 (IRF9); therefore, IFNT is unable to transactivate the classical JAK-STAT1 cell signaling pathway when binding to its receptor (IFNAR1/IFNAR2) and must transactivate alternate cell signaling pathways such as the mitogen-activated protein kinase pathway or other alternative cell signaling pathways reported by Plantanias [12] that have not been established for sheep. Restriction of expression of classical ISGs by ovine uterine LE/sGE is due to IFNT-induced expression of interferon regulatory factor 2 (IRF2), a potent transcriptional repressor, in those cells, but IRF2 is not expressed in uterine GE and stromal cells [10,13].

**Cows**. Bovine IFNT is secreted between Days 12 and 38 of pregnancy and activates mechanisms that prevent uterine secretion of luteolytic pulses of PGF [14]. As for ewes, neither exogenous E2 nor OXT stimulate the uterine release of pulsatile PGF, indicating lack of expression of *ESR1* and *OXTR* mRNAs in the endometria of pregnant as compared to cyclic cows, and in cows that received intra-uterine injections of ovine IFNT. In any event, bovine IFNT abrogates uterine production of luteolytic pulses of PGF.

**Goats**. Caprine IFNT is secreted between Days 16 and 21 of gestation to prevent pulsatile release of luteolytic PGF [15]. Intra-uterine injections of ovine IFNT in nanny goats extends CL lifespan.

**Figure 2:**
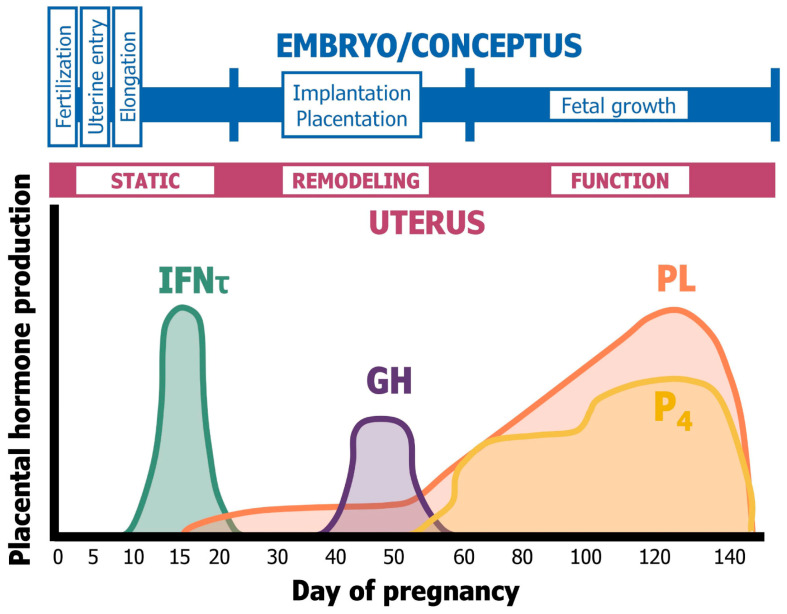
Servomechanism during pregnancy in sheep (see [6,19]). A servomechanism involves one hormone being essential for a subsequent hormone to exert its effect. In the ovine uterus, estrogen from ovarian follicles acts via its receptors to increase expression of receptors for progesterone (Prog) from the corpus luteum and Prog prepares the uterine environment for conceptus development and establishment of pregnancy. Interferon tau (IFNτ) not only signals for pregnancy recognition in sheep by abrogating the uterine luteolytic mechanism, but also its effects on the uterine epithelia are required for the uterine epithelial cells to respond to placental growth hormone (GH) and placental lactogen (PL). Placental lactogen and GH enhance proliferation and secretory activity of uterine glandular epithelial cells.

Establishment and maintenance of pregnancy in sheep require integration of endocrine and paracrine signals from the ovary, conceptus, and uterus [4,5,6,7]. Superficial implantation and placentation occur between Days 15 and 60 of pregnancy as the uterus grows and remodels to accommodate the development and growth of the conceptus in the last trimester of pregnancy [4]. In addition, caruncles and cotyledons develop and interdigitate to form placentomes that increase in vascularity and the intercaruncular endometrial glands grow substantially during pregnancy to secrete increasing amounts of histotroph, which is transported across the areolae of the chorioallantois into the fetal–placental circulation [16,17]. During gestation, endometrial gland hyperplasia occurs between Days 15 and 50 and is followed by hypertrophy for maximal production of histotroph after Day 60. During this period, the pregnant ovine uterus is exposed sequentially to E2, P4, IFNT, chorionic somatomammotropin hormone (CSH1), and placental growth hormone (GH) that regulate endometrial gland morphogenesis and differentiated functions [18,19]. This sequence of the effects of hormones, one being a prerequisite for actions of the next hormone, is referred to as a servomechanism (Figure 2). The binucleate cells of the chorion secrete CSH1 from Day 16 of pregnancy, which coincides with the initiation of expression of uterine milk proteins (UTMPs; serpin family of serine protease inhibitors) and secreted phosphoprotein 1 (SPP1, also called osteopontin, OPN), an extracellular matrix protein (ECM) that is an excellent marker of endometrial GE differentiation and secretory capacity [4,20]. In maternal serum, CSH1 is detectable by Day 50 and peaks between Days 120 and 130 of gestation [21] and can bind and transactivate cell signaling through both homodimers of PRLR and heterodimers of PRLR and GHR. In the ovine uterus, prolactin receptors (PRLRs) are expressed by GE [22,23]. Increasing levels of CSH1 are associated with hyperplasia and hypertrophy of uterine GE, as well as their increased production of UTMP, SPP1, and other components of histotroph that support the growth and development of the conceptus. Sequential exposure of the pregnant ovine endometrium to E2, P4, IFNT, CSH1, and placental GH constitutes a servomechanism that activates and maintains endometrial remodeling, secretory function, and uterine growth during gestation [7,9]. The chronic effect of P4 is to downregulate epithelial PGR to allow expression of proteins by uterine GE, while CSH1 and GH increase development of the endometrial glands and their expression of genes encoding for proteins such as stanniocalcin and SPP1 [19]. The net effect in ewes is a developmentally programmed sequence of events, mediated by specific paracrine-acting factors at the conceptus–endometrial interface that stimulate both intercaruncular endometrial remodeling and differentiated function of uterine GE for increased production of histotroph to support fetal–placental growth. Hormones including prolactin, P4 from the CL and placenta, CSH1, and GH, as well as glucocorticoids from the fetal adrenal and insulin from the pancreas, stimulate mammogenesis, lactogenesis, and uterine functions supportive of conceptus development in ruminants [19,24,25,26].

**Figure 3:**
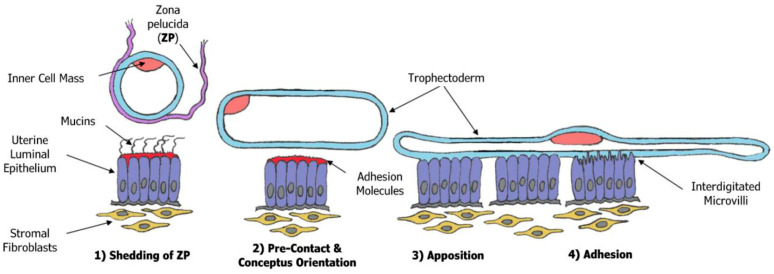
The stages of implantation of in sheep begins with the blastocyst shedding the zona pellucida (ZP) and then undergoing orientation and precontact with the uterine luminal epithelium. The next steps include apposition followed by adhesion of conceptus trophectoderm to uterine luminal epithelium during the peri-implantation period of pregnancy.

**Implantation of the blastocyst/conceptus and placentation in sheep.** The term “Implantation” is somewhat a misnomer for sheep, but it refers to the initial stages of placentation as trophectoderm cells of the filamentous conceptus come into apposition with and attach to the uterine LE progressively between Days 14 and 22 of gestation in both the caruncular and intercaruncular regions of the endometrium [4,27,28,29,30,31,32,33,34,35,36], as shown in Figure 3. The attachment cascade in sheep includes downregulation of mucin 1 (Muc1) in the uterine LE to “unmask” glycosylation-dependent cell adhesion molecule 1 (GlyCAM-1), galectin-15, and secreted phosphoprotein 1 (SPP1) for interaction with lectins and integrins [29]. Initial attachment may be mediated by GlyCAM-1 and galectin-15, and firm attachment by SPP1 [29]. P4 does not appear to decrease Muc1 expression on the apical surface of the uterine LE in sheep [36] while integrins are expressed constitutively by the uterine LE and conceptus trophectoderm during the peri-implantation period [4,29]. P4 increases expression of galectin-15 expression, which is further increased by IFNT [35]. P4 regulates expression of SPP1 by the uterine GE and it becomes a component of histotroph within the uterine lumen of pregnant ewes as early as Day 13, associated with loss of PGR by the uterine GE [36]. Immunoreactive SPP1 is present at the apical surfaces of the uterine LE, sLE, and GE and conceptus trophectoderm along with integrin subunits αv, α4, α5, β1, β3, and β5 that could contribute to the assembly of several SSP1 receptors including αvβ3, αvβ1, αvβ5, α4β1, and α5β1 heterodimers, which are expressed constitutively on the apical surfaces of the conceptus trophectoderm and endometrial LE [29]. Thus, SPP1 from the uterine GE binds integrin receptors expressed on the endometrial LE and conceptus trophectoderm for implantation and subsequent placentation in sheep [4,20,29]. Specifically, SPP1 binding to the αvβ3 integrin receptor induces assembly of integrin adhesion complexes (IACs), a prerequisite for the adhesion and migration of trophectoderm through the activation of the following: (1) P70S6K via crosstalk between FRAP1/MTOR and MAPK pathways; (2) MTOR, PI3K, MAPK3/MAPK1 (Erk1/2), and MAPK14 (p38) signaling to stimulate trophectoderm cell migration; and (3) IAC and myosin II motor activity to induce migration of conceptus trophectoderm cells [37]. Also, SPP1 binds integrins to form IACs that activate the MTORC2 pathway for cytoskeletal reorganization in both adhered and migrating oTr cells, and SPP1 acts with arginine to increase trophectoderm cell adhesion and migration [38]. Collectively, results indicate that osteopontin binds the αvβ3 integrin receptor to activate cell signaling pathways that act in concert to mediate the adhesion, migration, and cytoskeletal remodeling of conceptus trophectoderm cells essential for expansion and elongation of conceptuses and their attachment to the uterine LE for implantation [4].

**Synepitheliochorial placentation.** Synepitheliochorial placentation in sheep involves the fusion of the conceptus trophectoderm and uterine LE when there are both mononucleate trophectoderm cells and binucleate trophoblast giant cells (TGCs) represented in the ruminant placentae. In addition to mononucleate trophectoderm cells, TGCs differentiate from the mononucleate trophectoderm cells in concert with trophectoderm outgrowth during conceptus elongation. TGCs first appear between Days 14 and 16 of gestation in sheep conceptuses and comprise 15–20% of the trophectoderm during the apposition and attachment phases of implantation. TGCs migrate and fuse with individual uterine LE cells to form trinucleate syncytial cells beginning about Day 16 of pregnancy in sheep, thereby assimilating the endometrial LE. The syncytia of sheep enlarge due to TGC migration and fusion to form syncytial plaques between the uterine LE and placental chorion within placentomes that exist throughout pregnancy [39,40]. Recent immunohistochemical examination of the uterine–placental interface of both sheep and cows suggests there are limited periods of pregnancy in which placentation is syndesmochorial in which the LE is lost at the interface, and the formation of syncytia is more complex and varied than previously recognized [32,39,40,41].

During placentome development, highly branched villous placental folds termed cotyledons form by Day 30 of gestation in sheep [4,30]. The chorioallantoic villi are lined by syncytial plaques protruding into crypts in uterine caruncles (aglandular endometrium composed of stroma and overlying LE) to establish interdigitation between uterine and placental tissues by Day 40 of pregnancy. Within placentomes, the fetal–placental and maternal vasculatures are in close proximity for exchanging oxygen and nutrients with a high correlation between the placentomal mass and birthweight of fetuses. In contrast, epitheliochorial attachment of the uterine LE in interplacentomal areas of the uterus is characterized by the presence of an areolae associated with the opening of each uterine gland for uptake of components of histotroph from the uterine GE for transport into the fetal–placental vasculature [17,28,32,42].

***Integrin adhesion complexes (IACs)***. Integrin adhesion complexes (IACs) involve activated integrins rarely observed in vivo; however, large aggregations of IAC-associated proteins are present at the uterine–placental interface of sheep [17,29]. By Day 40 of pregnancy, punctate apical staining of integrin receptor subunits is present in the uterine LE and the conceptus trophectoderm is replaced by large aggregates of αv, α4, β1, and β5 subunits in the interplacentomal uterine LE and chorion only in the gravid uterine horn [17,28,29,31]. By Day 120 of pregnancy, IACs are detectable across most of the uterine–placental interface. Stromal cells in the gravid uterine horn exhibit upregulation of smooth muscle actin, desmin, and vimentin, indicative of myofibroblast differentiation. These stromal/myofibroblasts are surrounded by a connective tissue matrix that is more strain shielded due to crosslinking of the ECM in three dimensions (3D) compared to the complex forces at the maternal conceptus interface [31]. IAC assembly at the uterine–placental interface and within placentomes and stromal compartments reflects adaptation to increasing forces caused by the growing conceptus. Cooperative binding of multiple integrins to SPP1 at the endometrial–placental interface forms a strong adhesive mosaic to maintain a tight connection, increased tensile strength, and signaling activity between endometrial and placental surfaces characteristic of epitheliochorial placentation in sheep [17].

Mechanisms for establishing and maintaining pregnancy are highly variable across species. The pregnancy recognition signal may be either antiluteolytic or luteotropic. The focus of this review is on pregnancy in ruminants with sheep being the most studied species of ruminants [5]. In subprimate mammals such as ruminants (e.g., cows, sheep, and goats), the estrous cycle is regulated by hormones that lead to ovulation of an ovarian follicle that produces estradiol (E2) responsible for eliciting behavioral estrus, an ovulatory surge of LH, and LH-induced luteinization of granulosa and theca cells of the ovarian follicle that form the CL that produce P4. P4 is the essential hormone for the establishment and maintenance of pregnancy. Following a non-fertile mating during estrus, uterine epithelial cells respond to P4 to synthesize phospholipids that release arachidonic acid, in response to phospholipase A2, for the synthesis of PGF. The chronology of key events during the estrous cycle of ewes is as follows [5]: (1) P4 auto-downregulates its receptors in uterine epithelia 11 to 12 days after onset of estrus, which allows increases in expression of ESR1; (2) E2, acting via ESR1, induces expression of OXTR; (3) E2 induces expression of phospholipase 2 that cleaves arachidonic acid from phospholipids in uterine epithelia; prostaglandin synthase 2 (PTGS2) and PGF synthase generate PGF; and (4) pulsatile release of oxytocin (OXT) from the CL and posterior pituitary induces pulsatile release of PGF responsible for luteolysis. Luteolysis is the cessation of secretion of P4 and programmed cell death of luteal cells. PGF is the luteolytic hormone in most mammalian species. Accordingly, the conceptus (embryo and its extra-embryonic membranes) must produce an antiluteolyic signal, which is IFNT in domestic ruminants. IFNT acts via its receptors (IFNAR1/IFNAR2) on uterine epithelial cells, as demonstrated for sheep as a model ruminant species, to silence expression of ESR1, which abrogates the ability of E2 to upregulate expression of OXTR. Consequently, uterine epithelial cells do not express either ESR1 or OXTR, which prevents oxytocin-induced pulsatile secretion of PGF to allow maintenance of a fully functional CL that produces P4 required for the establishment and maintenance of pregnancy. In subprimate species, such as ruminants, hysterectomy results in cessation of estrous cycles since the uterus is the source of luteolytic PGF.

Ruminants differ from primates as women are unique in that their ovarian cycle is independent of the uterus and their ovarian cycle continues following hysterectomy [43]. Available evidence suggests that an intra-ovarian source of PGF is responsible for regression of the CL in women. In primates, chorionic gonadotropin (CG; hCG for humans) is the luteotropic hormone from trophoblast cells of the conceptus that acts via its receptor (LHCGR) on luteal cells to prevent luteolysis and to stimulate secretion of P4 during the period for the establishment and maintenance of pregnancy [44].

## 2. Interferon Tau (IFNT) and Its Various Roles

**Regulation of secretion of IFNT**. Interferon tau is secreted by the mononuclear trophoblast cells of sheep conceptuses from about Day 10, with maximum secretion between Days 14 and 16 of gestation, and then secretion of IFNT ceases around Day 21 of gestation. Thus, secretion of IFNT is developmentally regulated with gene expression being upregulated and downregulated in a specific temporal manner. Roberts et al. [45] proposed that the promoter element of the IFNT gene controls expression as it contains a v-ets avian erythroblastosis virus E26 oncogene homolog 2/ activator protein 1 (Ets-2/AP-1) enhancer element responsive to unidentified factors from the endometrium in response to P4 that transactivate the Ras and mitogen-activated protein kinase (MAPK) signal transduction pathway controlling transcription of IFNT by activating Ets-2. However, there are other possible explanations for the regulation of secretion of IFNT in a specific temporal pattern by ovine trophectoderm cells.

**Figure 4:**
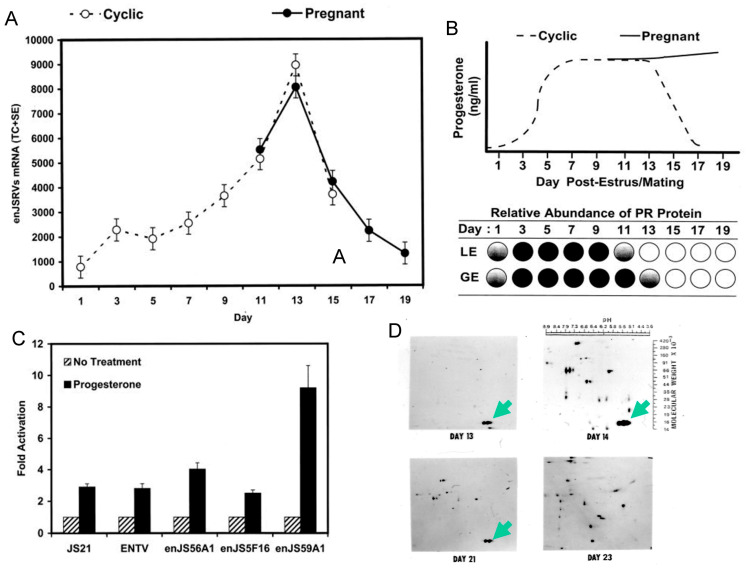
**Expression of endogenous Jaagsiekte retrovirus (enJSRV) and secretion of interferon tau (see [3,46,47,48,49,50,51,52]).** (**A**) The expression of enJSRV mRNA increases in both cyclic and pregnant ewes with greatest expression on about Day 13 after onset of estrus and then decreases in both cyclic and pregnant ewes. (**B**) The decrease in expression of enJSV occurs as concentrations of progesterone decrease and expression of receptors for progesterone (PR) decreases in uterine luminal epithelia after Day 11 and in uterine glandular epithelia after Day 13. (**C**) Expression of various isoforms of enJSRV is expressed in response to progesterone. (**D**) Secretion of interferon tau was reported originally based on its production using radiolabeled amino acids to be detected between Days 13 and 21 of gestation. A subsequent study determined that the amount of interferon tau produced per conceptus recovered on Days 8, 10, 12, 14, and 16 of gestation increased with days of gestation as mass of conceptus increased. These results, collectively, provide evidence that enJSRV affects the established temporal changes in expression of interferon tau.

There is the possibility that an endogenous retrovirus from the ovine uterus induces ovine trophectoderm cells to express IFNT (see Figure 4). The most abundantly expressed gene in the ovine genome is an endogenous retrovirus related to the Jaagsiekte Retrovirus (JSRV) that when in its exogenous form causes pulmonary carcinoma. Expression of the endogenous form of JSRV (enJSRV) by the uterine LE, sGE, and GE is regulated by P4 [46], receptors for enJSRV are expressed on ovine trophectoderm cells [47], and viral particles of enJSRV released into the uterine lumen are taken up by trophectoderm cells [48]. In pregnant ewes, expression of enJSRV increases between Days 11 and 13 and then decreases to Day 19 of gestation. This pattern of expression is very similar to increases and decreases in expression of IFNT by ovine trophectoderm cells during early pregnancy. Farin et al. [49] reported that expression of IFNT mRNA in ovine conceptuses increased from Day 11 to Day 13 before decreasing linearly to Day 23 of pregnancy, a pattern essentially identical to changes in expression of enJSRV mRNA in the endometrium of ewes. Because ovine conceptuses increase significantly in mass between Days 8 and 16, there is a corresponding increase in secretion of IFNT to Day 16 and then secretion of IFNT (IFNT secreted per conceptus per hour) decreases beyond Day 16 of gestation [5,50].

**Figure 5:**
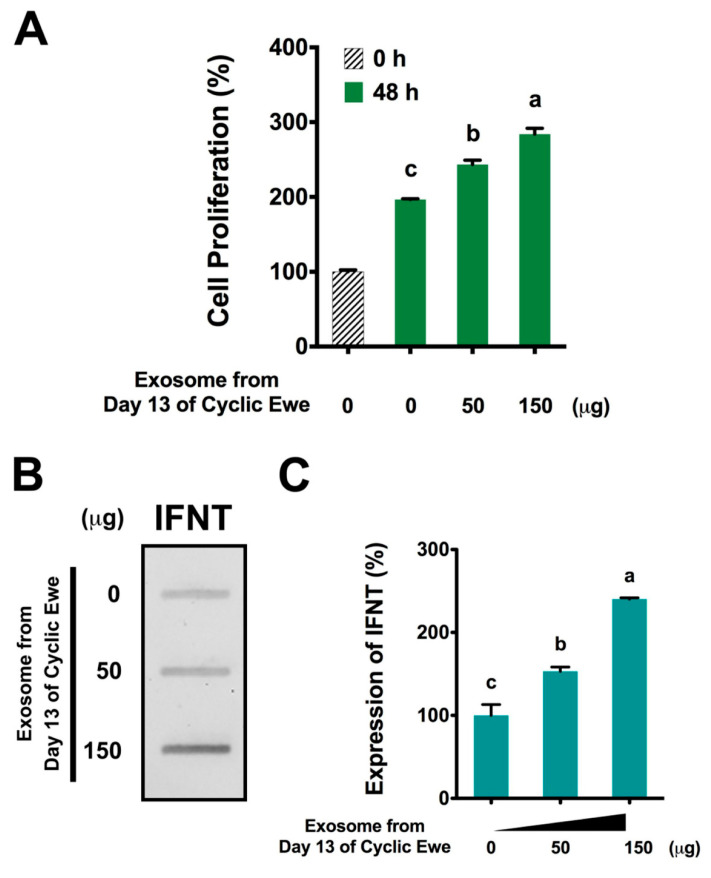
**Exosomes from uterine flushings from Day 13 of the estrous cycle stimulate proliferation of ovine trophectoderm cells and their secretion of interferon tau (IFNT) (see [51,52]).** The exosomes from the uterine flushings of cyclic ewes are taken up by trophectoderm cells, likely via toll-like receptors 7 and 8, or taken up by the trophectoderm cells by phagocytosis to increase cell proliferation by about 3-fold (**A**), IFNT in exosomes (**B**) and secretion of IFNT by trophectoderm cells by about 2.5-fold (**C**). The different letters among treatments with exosomes indicate significant (*p* < 0.01) differences.

Viruses and viral particles induce expression of type I interferons, such as IFNT, that interfere with the replication of viruses, thus the reason for naming those cytokines interferons. Does enJSRV induce and developmentally/temporally regulate expression of IFNT by ovine conceptus trophectoderm? Ruiz-Gonzales et al. [51] isolated exosomes from uterine flushings from cyclic and pregnant ewes and found that they contained mRNAs for the enJSRV-envelope protein, heat shock protein 70 (HSC70, a marker for exosomes), interleukins, and interferon regulatory factors (IRFs). Ovine trophectoderm cells proliferated and secreted IFNT in a dose-dependent manner in response to exosomes from cyclic ewes that would not be exposed to IFNT (Figure 5). The expression of mRNAs for cluster of differentiation 14 (CD14), cluster of differentiation 68 (CD68), interleukin-1 receptor-associated kinase 1 (IRAK-1), tumor necrosis factor receptor-associated factor 6 (TRAF6), interferon regulator factor 6 (IRF6), and IRF7 required for expression of toll-like receptor (TLR)-mediated expression of type 1 IFNs increases during the period of pregnancy recognition signaling in sheep. Further, exosomes recovered from the uterine lumen of cyclic ewes stimulated ovine trophectoderm cells to proliferate and secrete IFNT coordinately with regulation of TLR-mediated cell signaling. These results indicate that free and/or exosomal enJSRV taken up by trophectoderm cells via TLR induces secretion of IFNT in a manner that emulates the innate immune responses of macrophages and plasmacytoid dendritic cells to viral pathogens. Ovine conceptus trophectoderm expresses TLR7 and TLR8 between Days 13 and 16 of pregnancy when there is also expression of the envelope protein of enJSRV-Env by uterine epithelia [52]. Expression of the envelope protein of enJSRV in the trophectoderm declined from Day 13 to Day 16 of pregnancy. Morpholino antisense oligonucleotides (MAOs), when introduced into the uterine lumen of pregnant ewes on Day 8 of gestation, blocked translation of mRNAs for TLR7 and TLR8 to their respective proteins by the conceptus trophectoderm to assess the effects on conceptus development and secretion of IFNT on Day 16 of gestation. MAO-treated conceptuses were developmentally retarded, produced less IFNT, and had fewer TGCs compared with MAO-Controls. Moreover, expression of enJSRV-Env mRNA in MAO-TLR7 conceptuses was greater than that for MAO-Control and MAO-TLR8 conceptuses, but similar to MAO-TLR7/TLR8 conceptuses. These results support the hypothesis that TLR7 and TLR8 mediate pathways whereby enJSRV-Env regulates development of ovine trophectoderm cells during the peri-implantation period of pregnancy through stimulation of cell proliferation, secretion of IFNT, and formation of TGCs.

When considering results of the effects of enJSRV on ovine conceptus trophectoderm of ectodermal origin, trophectoderm cells and macrophages, of mesenchymal origin, share many characteristics including the secretion of interferons [53]. In addition, both trophectoderm cells and macrophages share the following characteristics: arginine and glutamine that are highly abundant in ruminant conceptuses are major mitogens [54,55]; both are phagocytic; both undergo syncytialization; both may be invasive; both express multiple toll-like receptors; and both express genes for cluster of differentiation 4 (CD4) and CD14, IgG receptor (FcR), non-specific esterase, granulocyte macrophage colony stimulating factor (GM-CSF), colony stimulating factor 1 (CSF1), interleukin 1 beta (IL1B), IL6, tumor necrosis factor alpha (TNFA), transforming growth factors (TGFs), platelet-derived growth factor (PDGF), and receptors for these cytokines. In utero, both trophectoderm cells and macrophages are responsive to factors from the uterine epithelia including the following: CSF1, GM-CSF, TNFA, TGFB, IL6, and leukemia inhibitory factor (LIF). The common characteristics and regulation of expression of trophectoderm cells and macrophages suggest that they play key roles in regulating local uterine immunity during pregnancy.

***Progesterone-induced and interferon-stimulated genes of uterine epithelial cells*.** IFNT is more than just the pregnancy recognition signal in ruminants. It is also responsible for variable phenotypes among cells of the uterine endometrium of sheep. IFNT upregulates expression of IFN-stimulated genes (ISGs), including classical ISGs, through STAT signaling in the endometrial stroma and GE of sheep and cows. In addition, nonclassical ISGs, including nutrient transporters, are upregulated in the uterine LE and sGE in response to P4 and further stimulated by IFNT in sheep [56]. IFNT induces expression of interferon regulatory factor 2 (IRF2) in the uterine LE and sGE, but not GE or stromal cells [13]. IRF2 silences transcription of ESR1 and OXTR to abrogate development of the luteolytic mechanism and prevent regression of the CL required for production of P4 for establishing and maintaining pregnancy [3,5]. The induction of IRF2 in the uterine LE and sGE also silences expression of classical ISGs in cells that do not express signal transduction and activator of transcription factors (STAT1 and STAT2), and interferon regulatory factor 9 (IRF9) required for cells to express classical ISGs. IFNT does not induce IRF2 in the uterine GE or stromal cells, which allows them to express classical ISGs. The uterine LE and sGE must respond to IFNT through a nonclassical cell signaling pathway as they do not express STAT1, STAT2, or IRF9. The alternate cell signaling pathway is yet to be defined. However, type I interferons can transactivate gene expression via non-canonical pathways including the mitogen-activated protein kinase (MAPK) pathway and phosphoinositide 3-kinase (PI3K)/mammalian target of rapamycine (mTOR) pathway [12]. The uterine LE and sGE respond to P4 and IFNT by increasing expression of genes for transport of nutrients such as amino acids and glucose, adhesion molecules such as galectin 15, cathepsins, cystatins for tissue remodeling, and hypoxia-inducible factor relevant to angiogenesis and survival of blastocysts in a hypoxic environment [57,58]. IFNT is also key to a servomechanism that allows uterine epithelia, particularly GE, to proliferate and to express genes in response to CSH1 and GH in sheep [6,19].

As noted earlier, P4 auto-downregulates its own receptor (PGR) in uterine epithelial cells between Days 11 and 12 of gestation, but expression of PGR is maintained in the uterine stromal cells of sheep [11]. Downregulation of expression of PGR in uterine epithelial cells is important because the effects of P4 on tissues are not specific to a cell type, but the effects of P4 on stromal cells to elicit secretion of a paracrine growth factor that acts on the overlying uterine epithelial cell in a paracrine manner allow for precise responses. Available evidence indicates that P4 acts on uterine stromal cells in sheep to induce expression of FGF10, FGF7, and HGF. FGF7 is expressed primarily by tunica intima of blood vessels, whereas FGF10 and HGF are expressed by stromal cells and their receptors, FGFR2IIIb and oncogene met (MET), respectively, are expressed by both uterine epithelia and the conceptus trophectoderm [8,9]. The uterine LE is lost during the latter part of the peri-implantation period (beginning on Day 17) in sheep to allow intimate contact between the trophectoderm and uterine stromal cells expressing FGF10 and HGF that may be important for epithelial cell functions and conceptus development [17,33,59]. FGF10 and HGF signal via the MAPK pathway, so if IFNT is using the non-canonical MAPK-pathway for signaling, this could explain the synergy between P4 and IFNT to induce and stimulate expression of a unique set of genes by the uterine LE and sGE.

**Interferon tau and the resolution of inflammation in the ovine uterus.** Prostaglandins and IFNT act in concert to regulate endometrial functions important for the growth and development of the conceptus during the peri-implantation period of pregnancy [60,61]. IFNT increases secretion of PGE2 and expression of mRNA for one of its receptors (PTGER2), but decreases expression of mRNA for the PGF receptor (PTGFR) in the endometrium. The same investigators detected expression of receptors for PGE2 (PTGER2 and PTGER4) and PGF (PTGFR) in the conceptus trophectoderm and endometrial epithelia of sheep during early pregnancy.

Sheep conceptuses from Day 14 of pregnancy produce similar amounts (ng/mg tissue) of PGF (32.1 ± 17.9) and PGE2 (12.3 ± 7.5) while Day 16 conceptuses secreted more (ng/mg tissue) PGF (9.0 ± 4.1) than PGE2 (0.9 ± 0.2) [62]. Sheep conceptuses from Day 14 secrete both PGF and PGE2, but more PGF than PGE2 [63,64]. Arosh et al. [65] reported that luteal cells in sheep produce PGF during luteolysis, but PGE during pregnancy in response to IFNT and that PGE from the uterus is transferred across the utero-ovarian vascular plexus to act as a luteal-protective hormone in the CL. Thus, PGE2 may be the more important prostaglandin during the establishment of pregnancy since IFNT decreases expression of PTGFR [60,61].

**IFNT and IL10.** Endometrial inflammation, characteristic of the implantation period of pregnancy in mammals, is resolved in eutherian mammals to allow pregnancies to be maintained until a second period of inflammation results in parturition. Hara et al. [66] reported that IFNT inhibits NLR family pyrin domain containing 3 (NLRP3) inflammasome-driven secretion of interleukin 1 beta (IL1B) in human macrophages that is IL10-dependent. Martal et al. [67] reported that administration of IFNT to mice on the day of implantation significantly decreased embryonic mortality while Assal-Meliani et al. [68] found immunosuppressive effects of both IFNT and interferon gamma (IFNG) to decrease spontaneous abortions in mice. Further, Chaouat et al. [69] discovered that IFNT prevents abortion in the CBA X DBA/2 mating combination in mice by inducing secretion of the immunosuppressive cytokine, IL10.

Interestingly, an investigation of the effects of IFNT on expression of IL4, IFNG, and IL10 by antigen-specific, CD4+ T cell lines derived from cattle revealed that IFNT upregulated IFN gamma secretion and steady-state levels of IFNG and IL4 mRNAs without affecting expression of IL10 mRNA [70]. Feng et al. [71] reported that IFNT regulates immune tolerance during pregnancy by promoting M2 macrophage polarization through inhibiting bta-miR-30b-5p-targeting suppressor of cytokine signaling (SOCS1) that inhibits the NFKB signaling pathway.

**Direct effects of interferon tau on the corpus luteum prevent luteolysis.** IFNT-mediated endocrine effects on the CL and other tissues of sheep were demonstrated in a series of studies in the laboratory of Professor Thomas Hansen [72,73]. Research in Professor Rina Meidan’s laboratory [74] clarified mechanisms whereby IFNT acts on the CL to ensure survival and secretion of P4. Her research compared transcriptomic profiles of the control CL from cyclic cows and the CL from cows on Day 18 of pregnancy. The luteolytic genes downregulated in the CL and endothelial cells of the CL of pregnant cows include endothelin-1 (EDN1), transforming growth factor-B1 (TGFB1), thrombospondins (THBSs) 1&2, and serpine-1 (SERPINE1). IFNT acts on large luteal cells to decrease expression of genes promoting apoptosis and anti-angiogenic genes for THBS1 and THBS2, as well as TGFBR1 and TGFBR2. Thus, IFNT enhances cell survival through suppressing cell death signals and upregulating pro-survival genes such as pentraxin 3 (PTX3), favoring a novel reciprocal inhibitory crosstalk between PTX3 and THBS1, favoring cell survival. However, IFNT does not directly affect secretion of P4 by the CL. Collectively, the effects of IFNT on the CL are to enhance cell survival and stabilize the vasculature of the CL to exert its antiluteolytic effects [74].

## 3. Unique Metabolic Pathways in Placentae of Ungulates

**Figure 6:**
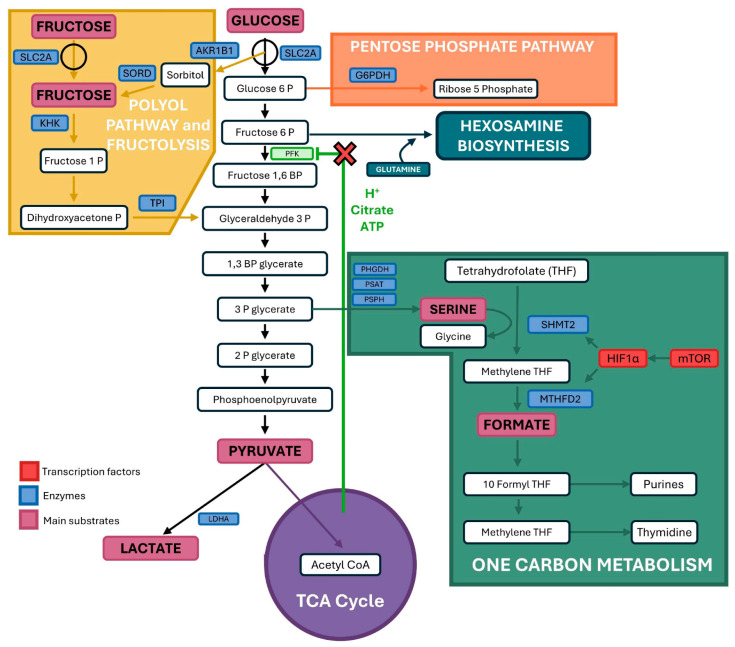
Fructose metabolism (fructolysis) in ovine conceptuses during the peri-implantation period of pregnancy [75,76]. Glucose transported into the uterine lumen and then into the conceptus trophectoderm by glucose transporters such as SLC2A3 is converted to sorbitol by AKR1B1 (aldose reductase). Sorbitol is converted to fructose by SORD (sorbitol dehydrogenase) and ketohexokinase (KHKA/KHKC) converts fructose to fructose-1-PO4 (F1P). F1P is metabolized to dihydroxyacetone phosphate (DHAP) and glyceraldehyde (GA) by aldolase B that are converted to glyceraldehyde-3-phosphate (GAP) by triosephosphate isomerase (TPI). GAP is converted to 1,3-bisphosphoglyerate by glyceraldehyde-3-phosphate dehydrogenase (1,3-BPG) that is metabolized to 3-phosphoglycerate (3PG). The latter is the carbon skeleton for synthesis of serine and glycine used to produce formate for synthesis of purines and pyrimidines for one-carbon metabolism. The 3PG can also be converted to 2-phosphoglycerate by phosphoglycerate mutase (PGM), then to phosphoenolpyruvate (PEP) by enolase and PEP is converted to pyruvate by pyruvate kinase (PK). In the presence of NADH, pyruvate is reduced to lactate by lactate dehydrogenase isoform A (LDHA), while LDHB favors oxidation of lactate to pyruvate in the presence of NAD+. Thus, LDH plays an important role in regulating cytosolic redox balance. Pyruvate may be oxidized to acetyl-CoA by pyruvate dehydrogenase (PDH), and acetyl-CoA can be used for synthesis of lipids, to regulate amino acid metabolism, or enter the TCA cycle. Note that HIF1A is upstream of KHKA/KHKC and that the fructolysis pathway is not inhibited by ATP, low pH, or citrate, as is the case for glycolysis. Importantly, metabolites of fructolysis are substrates for the pentose cycle, hexosamine biosynthesis pathway, one-carbon metabolism, and the TCA cycle. Further, the fructolysis pathway bypasses phosphofructokinase (PFK1) so it is not inhibited by high concentrations of ATP and citrate or low pH (see red X that indicates step at which inhibition occurs.

**Glycolysis versus fructolysis.** Humans and rodents have invasive types of implantation and either hemochorial (humans, monkeys, and rodents) or endotheliochorial (carnivores) placentae with close apposition between maternal blood and fetal blood for efficient transport of glucose metabolized via the glycolytic pathway with little or no conversion of glucose to fructose by the placenta. However, cetaceans (e.g., whales and dolphins) and ungulates (e.g., sheep, cows, and pigs) exhibit noninvasive implantation of the blastocyst into the uterine wall and have either epitheliochorial placentae (pigs, horses, cetaceans) or syndesmochorial placentae (cows, sheep, goats) with five or six layers of cells between maternal and fetal blood. In those species the placenta rapidly converts glucose to fructose that is metabolized via fructolysis (see [57,75]), as illustrated in Figure 6.

The unique advantage of fructolysis is that it bypasses the phosphofructokinase (PFK) step in metabolism of hexose sugars that can be inhibited by low pH, citrate, and ATP (see [57,58,75]). In sheep conceptuses, fructose is 15- to 30-fold more abundant than glucose in fetal fluids and plasma throughout pregnancy. Fructose is produced via the polyol pathway and is converted by ketohexokinase (KHK) to fructose-1 phosphate (F1P). F1P is metabolized via the fructolysis pathway that bypasses the regulatory step at PFK in glycolysis for unimpeded production of key substrates required for further metabolism via the major metabolic pathways that are the pentose cycle, hexosamine biosynthesis, one-carbon metabolism and serine synthesis, and tricarboxylic acid cycle. Fructolysis generates abundant amounts of lactate that may sustain expression of hypoxia-inducible factor alpha (HIF1A) by inhibiting its degradation. HIF1A is upstream in regulating expression of genes including vascular endothelial growth factor (VEGF), angiopoietins responsible for angiogenesis, and KHK [76,77].

Adaptation of the polyol pathway and fructolysis in the placenta involves the role of trophectoderm/chorioallantois to rapidly convert glucose to fructose that is sequestered in fetal fluids and plasma and cannot be transferred to the maternal vasculature. Then, fructose is converted to F1P and metabolized via the fructolysis pathway to provide substrates for further metabolism by the four major metabolic pathways required for growth and development of the conceptus. Fructolysis is unique to the period of pregnancy and conceptus development as fructose in fetal blood is excreted in urine during the first 24 to 48 h after birth to prevent development of insulin-insensitivity, and piglets fail to survive on synthetic diets containing only fructose, likely due to insufficient production of ATP in the brain causing damage to this organ. Further, there is increasing evidence that lactate, a product of fructose metabolism, transactivates its own receptor (HCAR1) to influence implantation and placentation, as well as sustain expression of HIF1A and its downstream genes such as KHK and VEGF [57,75,76].

**Figure 7:**
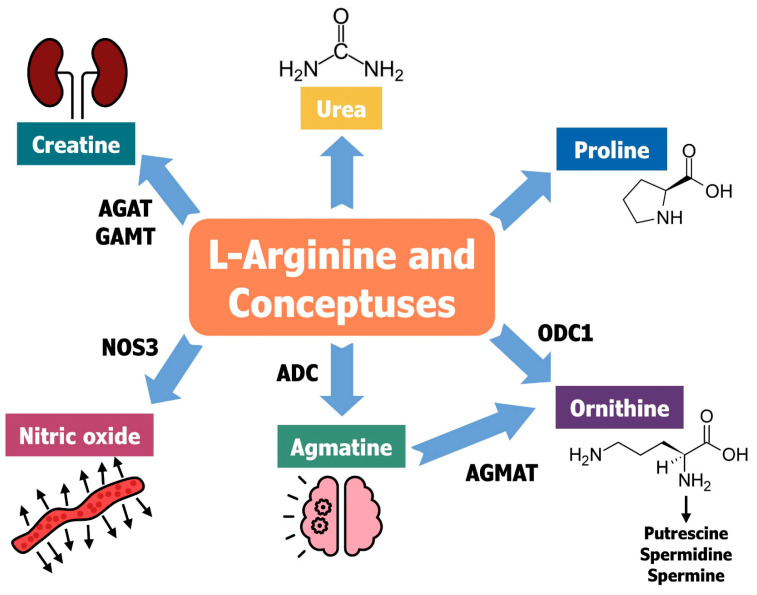
L-arginine (Arg) plays major roles in support of conceptus development (see [54,80,81,85]). Arg is required for synthesis of creatine and phosphocreatine critical for production of ATP, synthesis of nitric oxide for angiogenesis and vasodilation in the vascular system, generation of ornithine and agmatine required for synthesis of multi-functional polyamines, and synthesis of proline. Arg is also key to the urea cycle to prevent hyperammonemia. The arrows indicate the products of arginine metabolism.

**Multifaceted roles of arginine in ovine pregnancies**. Amino acids are critical for conceptus development because they are essential for protein synthesis and activation of cellular functions required for development, growth, and survival [78] (see Figure 7). Arginine (Arg) is a unique amino acid generally classified as a nonessential amino acid since it is synthesized in the body. However, it is now classified as a conditionally essential amino acid, meaning that it is synthesized in the body, but under physiological demands for rapid growth, pregnancy, and lactation, the body cannot make sufficient Arg, so dietary supplements are required for optimal performance by animals [79]. Arginine is required for the synthesis of nitric oxide (NO), polyamines (putrescine, spermidine, and spermine), and creatine that are critical for placental growth and conceptus development [80]. Arg is also metabolized to homoarginine, which increases progressively in maternal plasma during normal pregnancy in both sheep and humans [79]. Under normal feeding conditions for nonpregnant and late-pregnant ewes fed a diet with 12% crude protein, endogenous Arg synthesis from both glutamine and proline via the intestinal–renal axis in the host [81] contributes 65% and 68% of their total Arg requirements, respectively [82]. Of note, creatine production requires 40% and 36% of Arg utilized by nonpregnant and late-pregnant ewes, respectively [83]. Thus, there are high rates of Arg turnover in pregnant and nonpregnant ruminants. Arg also enhances insulin secretion by pancreatic β-cells and insulin-mediated anabolic effects for protein accretion in the conceptus [83,84].

A study to assess the roles of Arg in conceptus development and secretion of IFNT was conducted using an ovine trophectoderm (oTr1) cell line to determine responses to Arg, putrescine, and NO donors, as well as associated inhibitors [85]. Arg (0.2 mM, physiological concentrations in plasma) stimulated proliferation of oTr cells (2-fold), production of IFNT/cell 3.1-fold, and total protein per cell 1.5-fold, and increased abundances of phosphorylated tuberous sclerosis protein (p-TSC2) and phosphorylated mechanistic target of rapamycin (MTOR) 2.7- and 4.3-fold, respectively. Both putrescine and NO stimulated cell proliferation via activation of the TSC2-MTOR signaling cascade, whereas only putrescine increased IFNT production. These results indicated that Arg is essential for oTr1 cell proliferation and IFNT production via the NO-polyamine-TSC2-MTOR signaling pathways, particularly the pathway for polyamine biosynthesis [85]. Arg also stimulates secretion of IFNT via the NO and polyamine-TSC2-MTOR signaling pathway [86].

Stimulatory effects of NO on angiogenesis and vasodilation via activation of the cGMP pathway are well established. Therefore, the role of NO on ovine conceptus development was assessed in an in vivo study in which MAO-mediated knockdown of translation of nitric oxide synthase-3 (NOS3) mRNA in the conceptus trophectoderm (Tr) was performed [87]. Knockdown of NOS3 mRNA translation resulted in small, thin, and underdeveloped conceptuses, but normal production of IFNT. The amounts of ornithine and polyamines were less in uterine flushings, whereas the amounts of Arg, citrulline, ornithine, glutamine, glutamate, and polyamines were significantly less abundant in conceptuses, likely accounting for the failure of MAO-NOS3 conceptuses to develop normally. These results suggest that NOS3 is the key isozyme for NO production by ovine conceptus trophectoderm and that sufficient Arg in conceptus tissues for synthesis of polyamines is essential for conceptus survival and development [88].

Ovine blastocysts undergo rapid transitions from spherical to tubular and filamentous conceptuses in response to histotroph containing both Arg and SPP1. Arginine is transported into the uterine lumen and conceptus trophectoderm of sheep by solute carrier family member 7A1 (SLC7A1). Knockdown of translation of SLC7A1 mRNA using an MAO significantly inhibited transport of Arg into the uterine lumen, resulting in inhibition of conceptus elongation and abnormal conceptus morphology [89]. There were also significant decreases in abundances of ornithine decarboxylase and NOS3 proteins, citrulline, ornithine, and polyamines, accounting for retarded conceptus development. Thus, SLC7A1 is the key transporter of Arg by the conceptus trophectoderm, essential for ovine conceptus survival and development. A study with oTr1 cells revealed that migration and adhesion of oTr1 cells were stimulated by SPP1, an activator of placental ion transport. Arg alone increased cell migration and the combination of Arg and SPP1 had additive effects on migration and synergistic effects on adhesion of oTr1 cells. The cooperative effects of Arg and SPP1 were mediated by IAC-MTORC2-cytoskeletal reorganization and MAPK pathways. The combined effects of Arg and SPP1 on these cellular events are likely required for rapid elongation of ovine conceptuses during the peri-implantation period of pregnancy [90]. In a related study, knockdown of expression of integrin beta 3 (ITGB3) did not prevent elongation of sheep conceptuses, but decreased conceptus growth to Day 24 of gestation, and decreased expression of SPP1 and NOS3 [91]. Thus, in normal sheep pregnancies, both NOS3 and SPP1 positively influence development of the vasculature within the allantois required to transport nutrients from the endometrium in support of conceptus development and growth.

The polyamines (putrescine, spermine, and spermidine) regulate proliferation, differentiation, function, and death of cells including trophectoderm cells [92]. Arg is converted to ornithine by arginase type II and ornithine decarboxylase (ODC1) is the rate-controlling enzyme for biosynthesis of polyamines in mammals. An investigation of cellular functions of polyamines using in vivo knockdown of translation of mRNA for ODC1 in ovine conceptus trophectoderm using MAOs was conducted. The result was that one-half of the conceptuses was morphologically and functionally normal and the other one-half was morphologically and functionally abnormal. Survival of one-half of the conceptuses following knockdown of ODC1 in the conceptus trophectoderm was associated with their increased expression of arginine decarboxylase (ADC) that, in an alternate pathway to ODC1, converts arginine to agmatine, which is converted by agmatinase (AGMAT) to putrescine. Thus, the ADC/AGMAT biosynthesis pathway was upregulated in the absence of ODC1 to rescue the normal phenotype of one-half of the conceptuses [93]. These results were surprising since metabolism of Arg to agmatine via ADC and conversion of agmatine to polyamines by AGMAT is an alternative pathway long recognized in lower organisms but only reported for neurons and liver cells of some mammals (e.g., rats). Collectively, it appears that the majority of polyamine biosynthesis is via the ODC1-dependent pathway and that deficiencies in ODC1 increase activity of the rescue ADC-AGMAT-dependent pathway to rescue production of polyamines, at least in sheep. Nevertheless, both the ODC1 and alternative ADC-AGMAT pathways generate putrescine required for the development and survival of ovine conceptuses.

To further assess the significance of the ODC1 and ADC-AGMA pathways for biosynthesis of polyamines, a subsequent experiment used MAOs to inhibit translation of mRNAs for both ODC1 and ADC in ovine conceptuses [93]. Morphologies of MAO control, MAO-ODC1, and MAO-ADC conceptuses were normal whereas double knockdown of ODC1 and ADC resulted in only 33% of conceptuses appearing morphologically and functionally normal while 67% of the conceptuses were morphologically and functionally abnormal. In studies with MAOs, some conceptuses may survive due to insufficient uptake of MAO by the trophectoderm. Furthermore, MAO-ODC1:ADC normal conceptuses had greater tissue concentrations of agmatine, putrescine, and spermidine than MAO control conceptuses, while MAO-ODC1:ADC abnormal conceptuses had greater tissue concentrations of only agmatine. Uterine flushes from ewes with MAO-ODC1:ADC normal conceptuses had greater amounts of arginine, aspartate, tyrosine, citrulline, lysine, phenylalanine, isoleucine, leucine, and glutamine, while uterine flushes of ewes with MAO-ODC1:ADC abnormal conceptuses had lower amounts of putrescine, spermidine, spermine, alanine, aspartate, glutamine, tyrosine, phenylalanine, isoleucine, leucine, and lysine. The double knockdown of translation of ODC1 and ADC mRNAs was most detrimental to conceptus development and their production of IFNT was almost undetectable.

A subsequent experiment investigated the effects of agmatine on proliferation of oTr1 cells and the importance of the ADC/AGMAT alternative pathway for synthesis of polyamines in ovine conceptuses [94]. MAOs were used to inhibit translation of mRNAs for ODC1 alone, AGMAT alone, and their combination on conceptus development at Day 16 of gestation as in the previous experiment. Inhibition of translation of mRNAs for both ODC1 and AGMAT resulted in 22% of MAO-ODC1:MAO-AGMAT ewes having morphologically and functionally normal conceptuses while 78% of MAO-ODC1:MAO-AGMAT ewes had morphologically and functionally abnormal (not elongated and fragmented) conceptuses. As noted earlier, some 22% of conceptuses in the MAO-ODC1:MAO-AGMAT treated ewes appeared normal, possibly due to insufficient uptake of MAOs by trophectoderm cells. The pregnancy rate of 22% for MAO-ODC1:MAO-AGMAT ewes was less than for MAO-control (80%), MAO-ODC1 (75%), MAO-ADC (84%), and MAO-ODC1:MAO-ADC (44%) ewes. Inhibition of translation of both ODC1 and AGMAT mRNAs increased expression of mRNAs for ADC and transporters for polyamines (SLC22A1, SLC22A2, and SLC22A3), as well as abundances of agmatine, putrescine, spermidine, and spermine in conceptus tissue. However, MAO-ODC1:AGMAT ewes with abnormal conceptuses had greater abundances of agmatine, putrescine, and spermidine, but reduced amounts of spermine in uterine flushes, suggesting deficiencies in uptake by trophectoderm cells, and secretion of IFNT was significantly reduced by 50%.

## 4. Summary and Conclusions

This review highlights key morphological changes as the ruminant embryos develop into a blastocyst, elongate rapidly, signal through IFNT for pregnancy recognition, and undergo implantation and placentation. The novel features of pregnancy in ruminants are with respect to the roles of IFNT and the likely induction of secretion of IFNT by trophectoderm cells by enJSRV. IFNT not only abrogates the luteolytic mechanism, but induces expression of genes, such as those for transport of nutrients into the uterine lumen and many more associated with implantation and placentation. The unique use of fructose as the base carbohydrate for metabolism via fructolysis ensures uninhibited generation of substrates for further metabolism via the penose cycle, hexosamine biosynthesis pathway, one-carbon metabolism, and tricarboxylic acid cycle to meet demands for growth and development of the conceptus. Further, Arg is the key to synthesis of nitric oxide that promotes angiogenesis and vasculogenesis, polyamines that have many essential roles including those for stabilization of DNA and mRNA, and creatine that, as creatine-PO4, provides energy in the form of ATP required for growth and development of the conceptus. Both sheep and women have similar patterns of amino acid metabolism during pregnancy. Thus, IFNT is a novel pregnancy recognition signal, fructose is a unique metabolic substrate supporting all major metabolic pathways, and arginine is critical to multiple metabolic pathways required for conceptus development and survival. Collectively, those novel features of pregnancy in ruminants allow for successful establishment and maintenance of a successful pregnancy.

## Data Availability

Not applicable.
